# The Experience of Volunteers in Prisons in Portugal: A Qualitative Study

**DOI:** 10.3389/fpsyt.2021.778119

**Published:** 2022-01-04

**Authors:** Mónica Salselas, Mariana Pinto da Costa

**Affiliations:** ^1^Institute of Biomedical Sciences Abel Salazar (ICBAS), University of Porto, Porto, Portugal; ^2^Institute of Psychiatry, Psychology & Neuroscience, King's College London, London, United Kingdom; ^3^South London and Maudsley NHS Foundation Trust, London, United Kingdom

**Keywords:** volunteering, prisons, inmates, Portugal, qualitative research, experiences, social stigma

## Abstract

**Background:** Portugal is one of the countries that has a legal framework for volunteering, and there are different associations to support inmates through volunteering support. This volunteering can be beneficial for prisoners to address their social isolation and supporting them in the acquisition of skills and competencies to help them during their time in prison, but also beyond, supporting them in their resocialization and social reintegration in the community. However, little is known about the experiences of volunteers that provide such support to inmates.

**Methods:** Semi-structured interviews were conducted to explore the experiences and motivations of volunteers who interact with prisoners in the prison context of the three main cities in Portugal (Coimbra, Lisbon, and Porto). The interviews were audio-recorded, transcribed, and analyzed using the thematic analysis method.

**Results:** Thirty-nine prison volunteers agreed to participate in this study (*n* = 24 women, *n* = 15 men), with two to thirty years of experience of volunteering. The main themes emerging from the analysis were “Different motivations to volunteer”, “Volunteers” interactions with inmates”, “Volunteers” interactions with prison staff”, “Volunteering in prisons has an impact on volunteers”, “Volunteers” perception of helping inmates' and “More support to volunteering in prisons”.

**Conclusions:** Community volunteers who support prisoners can develop positive and trusting relationships with the inmates, despite its challenges. These findings can raise awareness of volunteering in prisons as a potentially helpful intervention, and call for further research to better explore its long-term impact.

## Introduction

Volunteering in Portugal has been done since pre-industrial times with carers providing support to families who required assistance, or driven by religious and spiritual beliefs that motivated people to do good and help others (1, 2). Before the appearance of the “Santas Casas da Misericórdia” (Holy Houses of Mercy) in the 15^th^ century, the “need to help” of the Portuguese population was answered through the provision of support in shelters, or through the provision of food from markets (1). Currently, volunteering can be done in various settings and targeting different groups, such as street volunteering, hospital volunteering or volunteering to support the elderly (3). Volunteering in prisons is neither the first option (1, 3–6), nor very common, and there is little awareness of it in Portugal (5, 7). People in the general population tend to be surprised when they learn that it is possible to volunteer in prison establishments, which are often the target of stigma (8).

The reality of volunteering in prisons has been explored across Europe in the project VOLPRIS (Prison Managing Volunteers in Europe) in five countries: Germany, Belgium, Poland, Portugal and Romania. The objective of VOLPRIS is to invest in the management of volunteering in the context of prisons, in order to positively impact not only the volunteers, but also the inmates' recidivism rates (7). This study conducted in seventy-nine prisons in these five countries reported data on volunteering in prisons: the importance of volunteering projects, the importance of the role of volunteers in the well-being of prisoners, the need for specific and adequate training, and the relationship between volunteers and prison staff (7). Some recommendations were made further to this study, such as: (i) promoting more research to demonstrate the diversity of volunteering projects in prisons and the impact that they have on social reintegration, (ii) improving the conditions for carrying out volunteering activities in prison establishments, and (iii) providing more information about volunteering opportunities in prison facilities (7).

Volunteers have an important effect on the inmates' attitudes, not only during their time in the prison, but also in the process of reintegrating inmates into society (9–11). Research conducted in Hong Kong (9) and the Netherlands (12) reported that volunteering in a prison context brings benefits not only to the volunteers, but also to the inmates themselves (9, 12). A study in the United States of America (USA) highlighted that whilst volunteers had positive attitudes toward prisoners and prison staff, the beginning of these interactions was marked by some mistrust (13). In contrast, according to a study carried out in Norway (14) the inmates showed positive attitudes toward prison staff and college students. Among the students, those who studied in the business economics area perceived prisoners in a more negative way than the healthcare students (14). This is similar to the results of a study in Australia (15), where medical students recognized the challenges and advantages of working in prison as a doctor, namely for the rejection of stereotypes. Studies carried out in Hong Kong (9), the Netherlands (12), Canada (16) and the USA (13) highlight that what led volunteers to become involved in prison volunteering contributed to the way they play their role as volunteer. The importance of visits made by volunteers, giving inmates opportunities to have different conversations and being away from the usual prison environment has also been highlighted (12).

In Portugal prison services also focus on the inmates' rehabilitation, using interventions to prepare the individuals for the moment of their release from prison (17). In this way, volunteers also play an important role in the resocialization process of inmates (17). To start volunteering in a prison environment, it is necessary to go through a selection process. This process involves two phases: (i) an initial selection by the organization who promotes the volunteering work (i.e., initial interview aimed at identifying the motivations, expectations and psychological characteristics of the person applying for the role of volunteer) and (ii) a final selection of the volunteer by the receiving organization (i.e., interview carried out in the prison by the volunteer manager technician and verification of the volunteer's profile) (18).

Portugal is one of the countries that has specific legislation for volunteering. The legal framework for volunteering (Law No. 71/98, of November 3^rd^) contains the main rights, duties and the institution principles that volunteers must follow (19). This legislation aims to promote and guarantee citizens the right to participate in the various activities, and to promote the freedom and flexibility associated with them (1, 19). The existence of this legal framework shows the importance of recognizing volunteering in Portugal, as well as the interest of the various entities promoting volunteering to support inmates, who are not in the habit of receiving many visitors, through various interventions (programs, activities and solidarity visits) which may help in combating their isolation (19). This law is an important instrument allowing volunteering to be qualified and socially recognized, by describing the legal rights of volunteers (19, 20). The previous legal diplomas that addressed this topic (i.e., Decree-Law No. 35108, of November 7^th^; Decree-Law No. 168/93, of May 11^th^) indicated the existence of solidarity projects that attract people to join volunteering (1), but the details about the rights and duties of volunteers, the definition of volunteering and the entities that promote volunteering were clearly defined in the Law No. 71/98, of November 3^rd^.

According to the Portuguese Annual Report of 2019 on Volunteering Activities of the Direção-Geral de Reinserção e Serviços Prisionais, there was an increase in solidarity visits, with 8,190 inmates receiving visits from volunteers and 1,968 people providing support as volunteers (5). However, between 2015 and 2019, volunteering in prisons has dropped across several intervention areas, including educational or training activities, cultural and artistic activities, and the promotion of sport and healthy lives (4, 5).

Since March 2020, due to the COVID pandemic, volunteering activities in the prison context have been suspended, as well as visits made by family members (21). However, within the remit of volunteering support in the context of prisons, the area of “Offer of Goods” experienced an increase during the pandemic. This was likely due to the suspension of visits made by family members since normally through them, the inmates received clothing and other essential goods (21).

The lack of knowledge in this area requires further attention. Thus, this study has aimed to: (i) explore the volunteers' motivations and the reasons that led them to volunteer in the prison environment; (ii) explore the interactions between volunteers and the inmates and prison staff and (iii) explore the individual impact that volunteering in prisons had on the lives of the volunteers. This study set out to investigate the research question: “*What are the motivations for, and the experiences of, volunteers who interact with inmates in a prison context?”*

## Methods

### Settings and Participants

**Figure 1:**
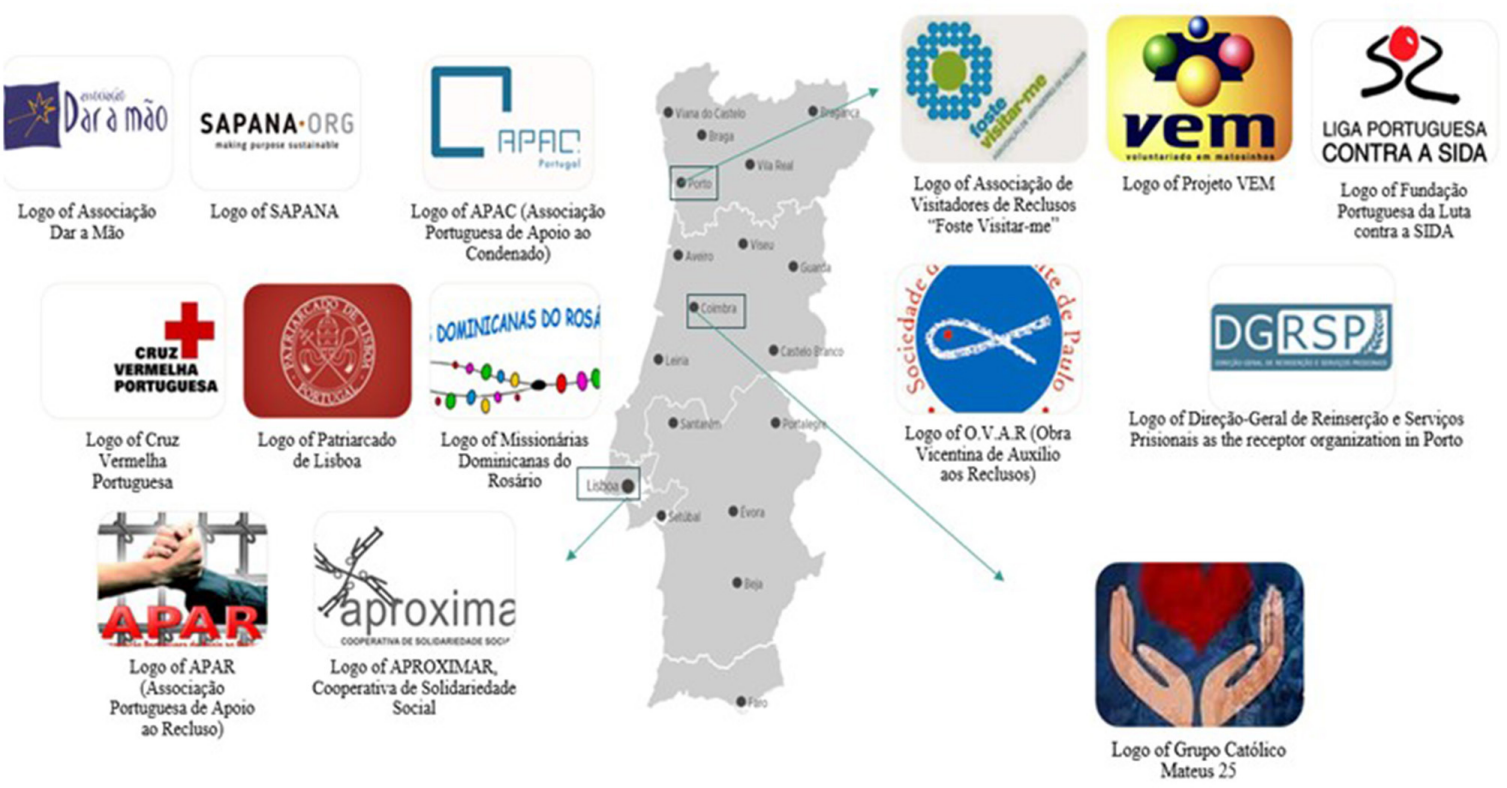
Volunteering associations that agreed to participate in the study.

Twenty-one organizations promoting volunteering in Portugal were contacted to carry out this study, of which fourteen agreed to participate. These organizations were located in the cities of Coimbra (*n* = 1), Lisbon (*n* = 8), and Porto (*n* = 5), and were selected based on their involvement in prison-based volunteering programmes (Figure 1).

The researcher (MS) contacted the representatives of the volunteering associations, providing them with information about the investigators, the purpose of this study, a brief description of the methods used and contact details for possible questions or concerns that could arise later. The participant information sheet with additional details about the study was sent via email to the respective representatives of each association, as well as a letter of support for the dissemination of the project to the volunteers of each association. It should be noted that until the time of the interviews, the authors did not know any of the participants. The only inclusion criterion considered was that the participants had volunteered in prisons (i.e., had participated in some activity or volunteering program with prisoners).

### Data Collection and Analysis

For this study, the semi-structured interview guide from Kort-Butler & Malone, 2014 (13) was translated into Portuguese, adapted and used to assist the interviews (Appendix 1). The researcher (MS) conducted the individual semi-structured interviews exploring the motivations that led to the involvement of participants in volunteering in prisons, the interactions that the volunteers established with the inmates and with the prison staff, and the impact of volunteers in the inmates and on themselves. Sociodemographic information was also collected (Appendix 2).

Due to the Covid pandemic, interviews were primarily planned to take place remotely or where possible, in person. The interviews were conducted by a female researcher (MS) and took place in a quiet location chosen by the participants. The data was analyzed through thematic analysis as outlined by Braun and Clarke (22) with the assistance of the QSR International Nvivo 12 software. The names of the volunteers were eliminated and replaced by numbers in order to protect their privacy. The initial codes were later organized and placed into themes. The themes were based on the scientific question, were again revised, and organized by the researchers (MS, who has a degree in criminology and MPC, who is a psychiatrist). The interviews were conducted in Portuguese, as well as the data analysis (Appendix 3). The sub-themes and themes as well as the quotes were translated into English by the researchers to be reported in this publication. The COREQ guidelines were followed for the study reporting (Appendix 4).

## Results

Forty-eight volunteers were contacted via email, and thirty-nine agreed to participate in this study. The volunteers interviewed from Coimbra, Lisbon and Porto consisted of twenty-four females and fifteen males, with an age that ranged between 26 to 76 years old. Their time of experience of volunteering in the prison context ranged from 6 to 10 years. None of the volunteers mentioned having served time in prison at any point in their lives. The interviews were conducted between March and July 2021, and ranged in duration from 17 min to 1 h and 46 min (with a mean of 52 min). The saturation point was reached at the end of the 39 interviews, since the information obtained in the last interview no longer included new data.

Only three interviews were conducted in person, the remaining 36 interviews were carried out through different platforms: Zoom (*n* = 23), Phone call (*n* = 7), WhatsApp (*n* = 4), Microsoft Teams (*n* = 1) and Google Meet (*n* = 1). The interviews were audio-recorded using the respective platform's recording system and later transcribed verbatim by the researcher (MS).

**Figure 2:**
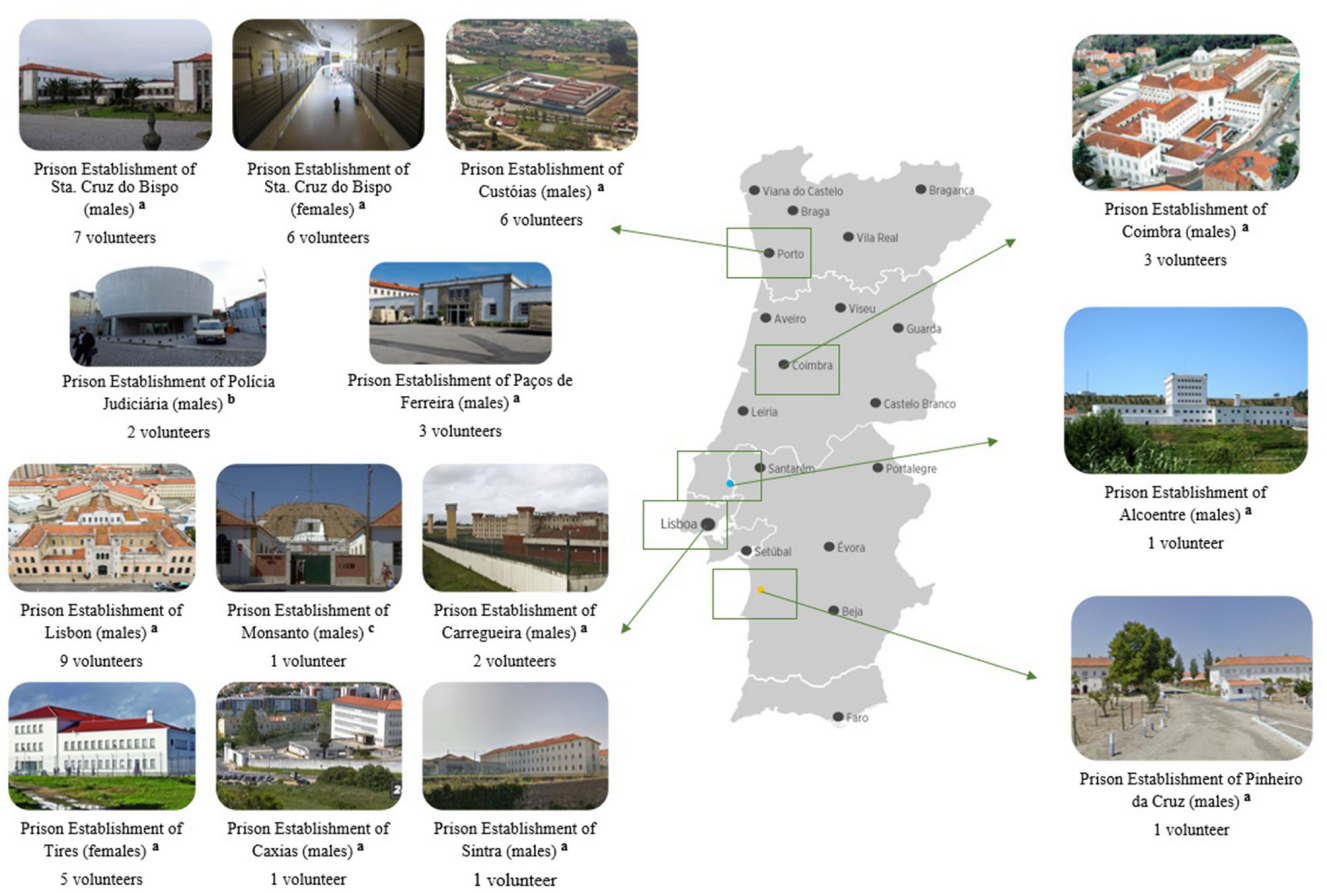
Prison establishments where volunteers supported inmates. ^a^Prison establishment with high security level. ^b^Prison establishment with high-medium security level. ^c^Prison establishment with high special security level.

Volunteers reported in which prisons they provided support to inmates, in a total of 14 prisons throughout the country. Figure 2 provides information about the prison establishments mentioned by the volunteers of where they volunteered, and how many volunteers supported each prison in this sample, with some volunteers supporting inmates from more than one prison (Figure 2).

There were six emergent themes in this data analysis: “*Different motivations to volunteer”, “Volunteers' interactions with inmates”, “Volunteers' interactions with prison staff”, “Volunteering in prisons has an impact on volunteers”, “Volunteers' perception of helping inmates”* and “*More support for volunteering in prisons”* (Table 1).

**Table 1:** Themes and subthemes.

**Themes**	**Different motivations to volunteer**	**Volunteers' interactions with inmates**	**Volunteers' interactions with prison staff**	**Volunteering in prisons has an impact on volunteers**	**Volunteers' perception of helping inmates**	**More support for volunteering in prisons**
Subthemes	To occupy their time Religious faith Need to help Previous experiences of volunteering Recommended by someone Opportunity to volunteer in prison emerged	Positive interaction with the inmates in prison Gaining trust with inmates Having better communication with the inmates Spending time out of prison during short-term outs	Prison staff initially suspicious of the volunteers Volunteers initially seen as obstacles by prison guards The volunteers' interactions with the prison guards improved with time and became cordial The prison environment was hard The volunteer managers were very accessible to the volunteers	Changing the volunteers' perspectives Force the volunteers to manage their expectations Relativization of volunteers' problems	Acquisition of skills Break in the routine A bridge between the inmates and their families A social bond with the outside world	Providing training and access to support to volunteers Careful selection of people who volunteer in prisons Improve prison conditions for carrying out volunteering activities Improve the relationship between volunteering associations and prison establishment Improve the image of the incarcerated population in society, and promote their reintegration

### Different Motivations to Volunteer

The volunteers described different reasons to become involved in volunteering in prisons (Table 2). Most volunteers had previous experience with other types of volunteering, although some volunteers chose to start volunteering in prisons to occupy their free time. Reasons ranged from religious faith, the need to help others, a recommendation made by someone, or the opportunity to volunteer in a prison, perceiving it as a way to get out of their comfort zone.

**Table 2:** Different motivations to volunteer quotes.

**Different motivations to volunteer**	
**To occupy their time**	“*I retired and had some availability. As I had free time, I ended up going to an initial meeting […]” (Volunteer 39)* “*[…] that's how I started, a little bit in order to help, to occupy my time in favor of something bigger*” *(Volunteer 10)*
**Religious faith**	“*Volunteering in a prison context arises, it is a consequence of my Catholic Faith” (Volunteer 16)* “*[…] it was a little bit also because of my religion because I have a Christian background […]” (Volunteer 10)*
**Need to help**	“*I always had this need to want to help other people” (Volunteer 37)* “*I felt there was a need to have a complementary commitment to society” (Volunteer 06)* “*Thinking that I could help in some way, that is, that I could give a better contribution to giving to people who were experiencing a moment of suffering” (Volunteer 02)*
**Previous experiences of volunteering**	“*Volunteering had already started earlier, but in other types of projects” (Volunteer 15)* “*Volunteering has always stayed with me, and I have always volunteered afterwards throughout my life” (Volunteer 31)* “*The world of prisons has always been present in my life, starting with my father [who worked as a doctor in prison] who told incredible stories of cases of inmates and then the volunteer work I did when I was 18 years old which marked me a lot too.” (Volunteer 01)*
**Recommendation of someone**	“*It's funny because it was a friend who came to me and said – look, I think I have a proposal that you'll like – […] and as I had a flexible work schedule, I decided to give a try.” (Volunteer 17)* “*It was at the suggestion of a friend of mine” (Volunteer 08)* “*[…] after I graduated, I went to work for the office of a lawyer who was the leader of a group of visitors in the prison establishment of Lisbon and he invited me to participate in that group.” (Volunteer 29)*
**Opportunity to volunteer in a prison emerged**	“*I had no motivation [specific], it was more that of leaving my comfort zone” (Volunteer 09)* “*It never crossed my mind to go into prison volunteering” (Volunteer 14)*

### To Occupy Their Time

Volunteers said that after retiring, they had more free time, and began volunteering as an option to occupy them.

### Religious Faith

Religious belief was a common motivation for volunteering. However, volunteers stated that they did not go to visit prisoners in order to impose their beliefs and values on the inmates, but to aid inmates whilst following prison rules.

### Need to Help

Volunteers described a commitment to society and a need to help the inmates. Throughout the interviews volunteers showed great concern for the prisoners.

### Previous Experiences of Volunteering

Previous experience of volunteering was common among volunteers. This involvement in volunteering led volunteers to be willing to continue their role as volunteers in settings or populations with whom they did not have experience before, such as in a prison context.

### Recommendation of Someone

The involvement in volunteering in the prison context for some volunteers emerged from recommendations made by friends or family or their mentor, either through knowledge of the associations, or through their own experience in volunteering.

### Opportunity to Volunteer in a Prison Emerged

Uncommonness contributes significantly to the lack of public awareness of prison volunteering opportunities. Some volunteers report that they had no prior intentions of volunteering in a prison. However, when this opportunity to volunteer in a prison emerged, volunteers appreciated leaving their comfort zone.

### Volunteers' Interactions With Inmates

By volunteering in a prison context, volunteers gain new perspectives through their interactions with inmates and the relationships they develop with them. This relationship had a positive evolution as volunteers maintain a repeated and constant presence, being able to communicate with prisoners without prejudice or judgement (Table 3).

**Table 3:** Volunteers' interactions with inmates quotes.

**Volunteers' interactions with inmates**	
Positive interaction with inmates in the prison	“*The relationship had to be based on truth, with honesty, without paternalism, without being top-down and that I realized early on, and I think I always tried to have that, so I think the relationship was always quite easy, with equal treatment” (Volunteer 25)* “*An attitude of honesty, loyalty is necessary, not to be little the trust they place in us at all, never in any way.” (Volunteer 33)*
Gaining trust with inmates	“*[…] an inmate who said that we were very important because by going there every week we showed that we had confidence in him and he said that the inmates had no confidence in anyone, neither them nor anyone else […]” (Volunteer 07)* “*The conversations I have with them, I often tell them my faults, it gives them this confidence that they are not abnormal but people who when further than they were supposed to go, but from now on is getting that and transform and then they start having this conversation” (Volunteer 33)*
Having better communication with the inmates	“*If there are questions, I mean that environment is always explosive because we're always finding people with very different characteristics and who are forced to live in those spaces and conditions, so I think calm is something that doesn't live inside these spaces, but we at least when we're there, we try to make thoughts fly elsewhere […]” (Volunteer 31)* “*The relationship that is established is therefore a relationship of knowledge, it is a person who is introduced to us and to whom we introduce ourselves, and from there we start a conversation that is marked by the space of the solidarity visit, it is a space of freedom as essential […]” (Volunteer 22)*
Spending time out of prison during short-term outs	“*I make the authorized visit to the exterior of the prison. […] Basically, we are responsible for those who have the right to make these precarious outings, we pick them up and go out one afternoon with them, we have lunch, and we stay until mid-afternoon with them. […] We have a great connection, we've known them for a few years.” (Volunteer 04)*

### Positive Interaction With Inmates in the Prison

The relationship that volunteers developed with prisoners is mostly positive. However, volunteers described the need to resort to conversation unblockers to break the ice as a way to start talking to the inmates and gain their confidence. Volunteers described individual characteristics that they deemed volunteers should have to reach out to inmates, such as the ability to listen, honesty, sincerity, equal treatment and the ability not to judge. Volunteers considered that having an open and unprejudiced attitude toward inmates facilitated these interactions.

### Gaining Trust With the Inmates

As in any other context, to create some kind of relationship it is necessary to build trust, which cannot be done overnight. With the prison population, the care and time taken to gain confidence is different, as inmates tend to be naturally suspicious.

### Having Better Communication With the Inmates

Volunteers presence in the prisons becomes frequent, which means that as the conversations gain more weight and the trust is built, the inmates end up mentioning life situations that they do not mention with their cellmates.

### Spending Time Out During Short-Term Outs

Some prisoners are allowed by the prison director to go outside the prison for a short period of time. During this period, and in certain situations, inmates may be accompanied by volunteers. In these cases, this monitoring is often done with the same inmates for some time, contributing to the establishment of a positive interaction between volunteers and inmates outside the prison.

### Volunteers' Interactions With Prison Staff

In addition to the contact that volunteers have with inmates throughout their voluntary work, they also gain knowledge about the prison system itself through the relationship they create with prison staff (Table 4).

**Table 4:** Volunteers' interactions with prison staff quotes.

**Volunteers' interactions with prison staff**	
Prison staff initially suspicious of the volunteers	“*The beginning was a bit troubled because they were very suspicious, cold people and a bit rigid” (Volunteer 38)* “*At first they were very suspicious [of the volunteers]” (Volunteer 39)*
Volunteers initially seen as obstacles by prison guards	“*[…] but in most of them, I'll be honest with you, what passes for us is that they don't see us as an asset, it's almost more of an obstacle.” (Volunteer 10)* “*[…] having a relationship with them that helps to undo this foreign body idea, but some have difficulty empathizing, some are easier” (Volunteer 03)*
The volunteers' interactions with the prison guards improved with time and were aimed as cordial	“*There was a very interesting evolution. Even in the first phase, we saw the guards almost as an obstacle to accessing the inmates and they also saw us with some disdain, with some reserve. Then we realized that when we go to visit everyone in the prison, we're going to visit the guards, the auxiliaries we come across, and all of them. […] So, we're going to visit the prison environment, we're going to visit the inmates, we're going to visit the guards who protect them and everyone else there included, and that completely changed the relationship. Over time, it changed [the relationship with the prison guards].” (Volunteer 06)* “*My relationship with the guards is a very respectful one” (Volunteer 34)* “*Our relationship tries to be as cordial and correct as possible, we try to be close to them” (Volunteer 03)*
The prison environment was hard	“*The one that impressed me the most was […] a high [special] security jail, where the inmates are locked 23 hours, you can't hear a fly, it's a horrible thing. I did interviews with inmates, only one agreed and then I also had a meeting with him alone. It was a bit complicated because they put me in a room with him that you can only leave when you press a button. So that was a little tense” (Volunteer 11)* “*I was with them [inmates] in a room where humidity was falling. […] water was running down the walls, so this is not a pleasant environment, let's say […]. I think there should be rooms to be with people, I didn't take off my coat inside, it was a complete ice.” (Volunteer 19)* “*[…] life inside the prison establishment is horrible. It's horrible, look, the prison corridors […] are immense, very wide, tall and on winter days, the fog that is outside is inside; the humidity that is outside is inside […]” (Volunteer 03)*
The volunteer managers were very accessible to the volunteers	“*With the techniques, with one or the others, friendship was even created, but I'm always staying in line here. A friendship relationship was created” (Volunteer 35)* “*I also have a positive relationship with the technicians.” (Volunteer 37)* “*[…] they are fantastic and even when we need something to enter material for the sessions, we are always careful not to take things that are dangerous, but I don't think I remember ever asking for anything that has been denied.” (Volunteer 15)* “*It was great, it was a very good relationship, and she was a very interested person. I spoke with her, and we always combined things with a view to improving what was possible to improve the body of the choir. […]” (Volunteer 36)*

### Prison Staff Initially Suspicious of the Volunteers

Before there is any interaction with the inmates, volunteers must have contact with prison guards, particularly when entering and leaving the prison. This first contact was not always positively described. At an early stage, volunteers described this interaction as cold, with some suspiciousness from prison guards, who were distant and sometimes even posed obstacles to volunteers when entering in the prison establishment.

### Volunteers Initially Seen as Obstacles by Prison Guards

At the beginning, due to the distance that the guards kept from the volunteers and the strangeness of their presence in the prison, some volunteers said that they felt perceived as obstacles by the prison guards. They also felt that they could be hindering the work performed by the prison guards themselves.

### The Volunteers' Interactions With Prison Guards Improved With Time and Became Cordial

The interactions between volunteers and prison guards evolved over time, and the initial problems mentioned no longer existed. Volunteers stated that after the first volunteering sessions, the prison guards became more accessible, increasingly trusting the volunteers, being positive in their interactions, and treating them with cordiality and mutual respect and, that they were more satisfied and committed to continue volunteering.

### The Prison Environment Was Hard

Volunteers described the environment within the prison as hard. The structure and buildings of prison establishments are old, and they have few conditions for proper spaces adequate for volunteering activities.

### The Volunteer Managers Were Very Accessible to the Volunteers

Although contact in the prison was mostly between the inmates and prison guards, the volunteers also maintained contact with the volunteer managers, the technicians who oversee the volunteering work, although less frequently. This interaction established with the volunteer managers was described as very positive with the technicians showing themselves to be quite accessible to the volunteers.

### Volunteering in Prisons Has an Impact on Volunteers

Volunteering in a prison context is a less known reality in the general population in Portugal. However, as the contact with this reality increases and, consequently, the contact with the inmate population, the impact that this volunteering causes in the lives of volunteers increases (Table 5).

**Table 5:** Volunteering in prisons has an impact on volunteers' quotes.

**Volunteering in prisons has an impact on volunteers**	
Changing the volunteers ‘perspectives	“*I realize that my reality is not the only one and it is always known as much as any experience outside its context does. That's why I think that's it, it gives greater social opening, I'm more aware of the realities that exist and the situations of injustice that also exist” (Volunteer 25)* “*Every contact with a reality different from ours helps us to create the possibility of empathy and, I don't know, opens up a bit of the world and our heads to understand other realities” (Volunteer 25)*
Forced the volunteers to manage their expectations	“*That expectation of if it is possible to collaborate for a person to reintegrate into society, we are always with this expectation, although that is not what we expect” (Volunteer 08)* “*Go and wait for nothing, go and just be with them and nothing else. […] It's not expecting anything from them but giving them a different morning” (Volunteer 07)*
Relativization of volunteers' problems	“*We relativize much more certain things that happen to us in life” (Volunteer 26)* “*We put things in the right priority. […] We give more value to exactly what we have and what we normally take for granted” (Volunteer 15)*

### Changing the Volunteers' Perspectives

The contact with other realities different from the one that the volunteers lived in made them gain other perspectives. They described realizing that there are other realities outside their professional and personal environment that, until they had contact with the inmates and heard their stories, they were unaware of.

### Forcing the Volunteers to Manage Their Expectations

Volunteers said that managing their own expectations was one of their biggest challenges. They recognized that they could only help in very few things because the inmate's reality does not depend on the volunteers.

### Relativization of Volunteers' Problems

With the experience of volunteering in a prison context, the volunteers said that they ended up relativizing certain problems. This relativization led them, in a way, to reformulate their priorities, not taking things for granted in their own lives.

### Volunteers' Perception of Helping Inmates

Volunteers perceived volunteering in prisons as something positive in the lives of inmates, bringing them various benefits (Table 6).

**Table 6:** Volunteers' perception of helping inmates' quotes.

**Volunteers' perception of helping inmates**	
Acquisition of skills	“*Relieving tension, being busy. Some learn professions and how to be useful to society through those contacts of the workshops that sell what they are producing.” (Volunteer 34)* “*The tools help to establish dialogue, share ideas, until they get to know each other better” (Volunteer 28)* “*We tried to take some varied activities, from texts to something more practical for them to do too, for them to participate […]” (Volunteer 24)* “*We always prepared a theme, a text, a dynamic to involve them and help them share, but individual conversation was also very important.” (Volunteer 23)*
Break in the routine	“*It helps to get through that time and it's constructive, they're constructive. They get used to being in a group, having schedules, having discipline. The day-to-day routines and the weeks somehow, our projects were there breaking some routines” (Volunteer 17)* “*In order to give them some conviviality, some coexistence with the outside world that they did not have, not even the family visited them” (Volunteer 08)* “*It is important to contribute to making a little difference in their day” (Volunteer37)*
A bridge between the inmates and their families	“*We often end up making the contact with the families and taking, or helping, family members to visit […] and this happens, sometimes we sponsor the coming of a family […] from Guarda or from another point of the country, so that they can come and visit the inmate that is in prison establishment of Tires.” (Volunteer 17)* “*I was never afraid because I don't have reasons, […] there is an ongoing conversation, and we usually collect phone numbers to call the families.” (Volunteer 14)* “*We do a little this bridge between the inmates inside and the family outside and this is also very rewarding and it's something that doesn't cost us anything. Whatever we can do that is basic and harmless, we always try to help with the knowledge of the prison.” (Volunteer 01)*
A social bond with the outside world	“*We are someone who comes from the outside and brings something new. […] It is important that they have someone to talk to, someone outside the system” (Volunteer 02)* “*We are a little bit the window that opens for them, the window that comes from the outside and we bring there a little bit of encouragement, of hope, of trust” (Volunteer 01)* “*[…] in order to give them some conviviality, some coexistence with the outside world that they did not have, nor did the family visit them” (Volunteer 08)*

### Acquisition of Skills

Volunteers mentioned the importance of volunteering programs and activities, as these programs aim to teach inmates skills that could be useful for them in the future.

### Break in Routine

To combat the routines in prison, these interactions with volunteers provide new opportunities, new routines, and new schedules because the inmates are already counting on activities or visits on those days.

### A Bridge Between the Inmates and Their Families

Volunteers end up being the contact between inmates and the outside world, particularly with families. Whenever possible and with the knowledge of the prison's management, volunteers could contact the inmates' families and even help with transporting so that families could visit their inmates in prison. Inmates are not always in a prison establishment close to their residence area, which sometimes makes it difficult for families to bear the costs of long journeys.

### A Social Bond With the Outside World

The regular presence of volunteers in front of inmates contributes to the continued existence of social bonds despite inmates' confinement. Through visits and activities, volunteers end up having time with the inmates, where they can speak openly and without judgment, promoting communication and combating the isolation of inmates.

### More Support to Volunteering in Prisons

Volunteers made some recommendations to improve the reality of volunteering in a prison context. These suggestions focus especially on maintaining a demanding training programme. Furthermore, volunteers also suggested that volunteering in this context be considered as a form of reintegration into society that can go beyond the prison establishment (Table 7).

**Table 7:** More support to volunteering in prisons quotes.

**More support to volunteering in prisons**	
Providing training and access to support to volunteers	“*Formations I think are very important, which is to give us the strength to go, to believe, to feel renewed in helping” (Volunteer 14)* “*The best way to improve volunteer activity is to maintain critical and ongoing training […]” (Volunteer 06)* “*It was more the support they give us from the establishment. I think from them we don't have as much support as we should have” (Volunteer 10)*
Careful selection of people who volunteer in prisons	“*I recommended volunteering in the prison context only to people who have a set of very specific characteristics. […] You have to be persistent, motivated individual with an extraordinary ability to listen.” (Volunteer 22)* “*The volunteer has to have certain characteristics very strong to face such a challenge. Above all, knowing how to listen, not making judgments, […] give opinion when necessary, keeping absolute secrecy, not entering into legal fields, they seem very simple things but are not for many people” (Volunteer 35)* “*You have to have a profile, you know?” We tend to accept people with some motor skills, who don't get too emotionally involved with the inmates, who are compliant, who are faithful, we're not exactly doing a job that anyone else can handle.” (Volunteer 06)*
Improve prison conditions for carrying out volunteering activities	“*[…] in terms of facilities for the performance of activities, therefore there should be an institutional effort by the General Management to create, within the physical possibilities, conditions so that this volunteering could be done in a more fruitful way. Volunteering […] is an external reality, has to adapt and adaptations and adjustments have to be made, and there are things that sometimes would benefit if they could be done in their own space and with proper conditions so there is a physical differentiation, that being a space of freedom within a space of reclusion.” (Volunteer 22)*
Improve the relationship between volunteering associations and prison establishment	“*I am convinced that the relationship with entities in the prison system is important in volunteering in the prison context. […] being able to break this barrier, in the sense of creating a good environment between prison entities and volunteer work, that I think was something to be done. This relationship with the prison structure is important, that would be the advice I would give - bet on the relationship with the prison structure.” (Volunteer 08)* “*I think volunteering should put an end to the “chapels”, there should be no “chapels”, yes I have my organization and you have yours. This level of mutual help between associations as I see it, does not exist. If there was a union of volunteers […] maybe we could change certain rules so that more dignified people, more human, would come out.” (Volunteer 33)* “*Greater flexibility in terms of accreditation, the admission process for volunteers and spiritual assistant collaborators who are not really volunteer visitors is very time-consuming and this is sometimes discouraging.” (Volunteer 22)*
Improve the image of the incarcerated population in society, and promote their reintegration	“*I would very much like the prison system to look at volunteering as a vehicle for reintegration. […] I would like volunteering in general to be seen as another arm to help these people with their reintegration and sometimes it's not even reintegration, it's integrating them for the first time in life” (Volunteer 31)* “*Extended volunteering to post-prison” (Volunteer 02)* “*Given the prison reality, it is very important that this happens and that there is interaction between society and incarcerated society because it is really a section of the population that is totally isolated and doesn't have [contact], at least I've never had contact with it, it a reality completely unfamiliar to the normal, so it is inevitable that the stigma lasts forever and that a person leaves and does not have opportunities.” (Volunteer 25)*

### Providing Training and Access to Support to Volunteers

Volunteers mentioned the importance of having detailed and ongoing training before entering the prison. Almost all volunteers received training before starting volunteering inside the prison establishment. In this setting, it is necessary to bear in mind the rules that exist and, to avoid future problems, volunteers should be mindful, aware, and prepared to possible situations that might happen so that they know how to deal with them in the best way.

### Careful Selection of People Who Volunteer in Prisons

Volunteers considered that there should be a careful selection of volunteers with the necessary characteristics for someone to volunteer in a prison and to be able to communicate with the inmates. Prisons were described as a difficult and heavy environment, not everyone has the necessary qualities, nor can they adapt to the prison environment.

### Improve Prison Conditions for Carrying Out Volunteering Activities

Volunteers referred to the improvement of conditions in places where volunteering activities take place. Prison establishments are normally places with a hostile environment and, to facilitate this volunteering, a favorable atmosphere should be created during these activities for inmates to abstract.

### Improve the Relationship Between Volunteering Associations and Prison Establishment

Volunteers mentioned the importance of having a good relationship between volunteering associations and prison establishments so that the surrounding environment is one of union and organization. Besides, the relationship among the volunteering associations themselves is always important, facilitating dialogue and cooperation between them.

### Improve the Image of the Incarcerated Population in Society, and Promote Their Reintegration

Volunteers recommended volunteering as a form of reintegration for inmates in the prison, but also to extend volunteering beyond the prison context to the moment of departure. Some volunteers supported greater contact with the prison population as a way to reduce the stigma associated with inmates and normalize their reality.

## Discussion

### Key Findings

Volunteers emphasized the importance of adequate training in the preparation for volunteering in prisons, and that the volunteers should be carefully selected. Without this, boundaries can be unclearly defined, potentially leading to problems such as the emotional involvement with an inmate, manipulation or even loaning money.

The importance of the activities that are carried out with the inmates was also highlighted, since these are aiming to support prisoners to gain skills and competencies that will be useful for their reintegration process outside prison, to stimulate a process of introspection and establish short, medium, and long-term goals.

Volunteers perceive their role as impactful in the inmates, but also in the surrounding prison environment. After gaining the inmates' trust, it was possible for inmates to talk about matters with the volunteers that they would not want to talk to cellmates.

### Strengths and Limitations

As far as we know, this is the first study in Portugal on volunteering in prisons. The study covered multiple areas: volunteers' motivations, the interactions that volunteers established with inmates and with professional staff, and the impact that this volunteering had on the lives of volunteers. Therefore, these findings add to a very limited literature base and hopefully set grounds for further work. The geographic coverage of this study is also a strength, as the volunteers belong to the main cities in Portugal providing us rich and detailed information about the phenomena.

The study has however some limitations. Firstly, the sample covers primarily volunteering associations based in urban areas and not in rural areas. Secondly, the volunteers were not directly asked if at any time they served a sentence in a prison or if they had a family member who has been incarcerated, which limits the understanding of the characteristics of these volunteers, and how individual factors may play a role in their motivation to volunteer in the prison setting. Finally, the perspectives in this study were only based on the volunteers' perspectives, and therefore the perception of inmates of these same interactions has not been investigated in this study.

### Comparison With the Literature

In our study, volunteers in the prison context in Portugal showed a very positive attitude toward inmates, demonstrating an easy attitude in their presence, without fear. This positive attitude toward inmates has also been found in research in other countries such as with volunteers in Canada, where the outcomes of a voluntary visits programme focusing on benefits to inmates, volunteers and prison staff were positive (16). Volunteering visits were beneficial not only for inmates, since these gave them the opportunity to talk safely and adopt a more optimistic view of the future, but also for the volunteers themselves since they felt more appreciative of their own quality of life (12, 16).

In this study, volunteers in prisons were mostly motivated by the greater availability and time in their lives, past experiences, and the need to help others. These same motivations were described in other research conducted in Southern states in the USA, where volunteers expressed their personal beliefs as one of the reasons for volunteering, sharing their blessing and values to the inmates, the commitment they felt toward volunteering and toward the volunteers themselves also contributed to their involvement in volunteering in the prison context (13, 23). Similarly, another study conducted in the state of Minnesota in the USA acknowledged that when it comes to volunteering, volunteers feel the need to help others and express their values and beliefs as a way to show their concern with others (24). Likewise, in another study conducted in prisons in the state of Mississippi in the USA, most chaplains who were involved in the religious programs understood that their function was primarily to support, encourage and share their faith with the inmates. Chaplains' efforts were to use their presence to transmit messages of hope to inmates at times when they were confronted with the negativity and the difficulties of the prison environment (11).

Volunteers in this study said that volunteering in prisons is important, and that it can bring benefits to the inmates and to themselves. This perception was previously described in another study conducted in the state of Florida in the USA, where it was found that volunteer visits can have a positive influence on the inmates, and influence their attitude while serving their sentence, contributing to the establishment of social relationships during incarceration (25, 26). A similar finding was reported in another study conducted in Hong Kong, emphasizing the importance of the role of volunteers during incarceration, where volunteers help inmates to build and improve their personal, family, and social relationships so that they can successfully re-enter in the society (10, 11).

### Implications of the Findings for Practice, Policies, and Research

Our study shows that some actions are required to improve the volunteering in the prison context in Portugal, namely: (i) providing training and access to support to volunteers who volunteer in prisons, so that volunteers know from the beginning what they can and cannot do, always complying with the necessary rules and avoiding any type of complication within the prison establishment that could jeopardize their safety, the safety of inmates and even the professional staff, (ii) improve the organization and the cooperation between the volunteering organizations and the prison establishment, providing more support from the prison establishment by improving conditions within the prison, so that volunteer activities can take place as naturally as possible and trying to achieve new areas of intervention within prisons, for example providing administrative support to technicians through monitoring, organizing processes and activities, (iii) improve the relationship that exists between society and the prison population as a way of contributing to reintegration and reducing the social stigma that these people face after serving their sentence and (iv) providing greater financial support to entities to be able to support the costs or facilitate more resources for the development of volunteering opportunities. According to the Portuguese legal framework of volunteering (Law No. 71/98, of November 3^rd^), one of the rights of volunteers is the possibility of having voluntary social insurance (19). Therefore, in order to be able to support insurance, transport expenses and materials for activities, it is necessary that the entities have the required financial capacity (17).

Further research could investigate the perception of the inmates and the prison staff of volunteering in prisons, assess the proportion of people who volunteer in prisons and conduct follow-up studies to assess the long-term impact of volunteering in prisons for the inmates and for the volunteers themselves. Since the environment of different prisons may vary depending on their size or security level, future research should explore the differences in the provision of volunteering according to their level of security (low-security vs. high security) and prison size (small institutions with a few hundreds of inmates vs. larger jails with thousands of inmates).

## Conclusions

This study outlines the volunteers' experiences of volunteering in prisons in Portugal, providing more information about this understudied area. Volunteers' motivations to support inmates in a prison vary from a wish to occupy their time and help other people, to having previous experiences of volunteering in other contexts or being encouraged by someone to volunteering in this setting. These findings show that, despite some challenges, the experiences of volunteers in the prison context in Portugal were largely positive. In fact, volunteers perceived their role as impactful to the inmates during their time in prison, supporting them in their reintegration into society, after serving their sentence, and also in themselves, changing their perspectives, their expectations and making volunteers relativize their own problems.

## Data Availability Statement

The original contributions presented in the study are included in the article/Supplementary Material. Further inquiries can be directed to the corresponding authors.

## Ethics Statement

This study was reviewed and approved by CHUP/ICBAS Ethics Committee of the Institute of Biomedical Sciences Abel Salazar (ICBAS) at the University of Porto - ref: 2021/CE/P005 (P345/CETI/ICBAS). The participants provided their written informed consent to participate in this study.

## Author Contributions

MS and MPC conceived the study, analyzed the results, and wrote the paper. MS made all the contacts with the volunteers, performed the interviews, and led the analytic process. All authors contributed to the article and approved the submitted version.

## Conflict of Interest

The authors declare that the research was conducted in the absence of any commercial or financial relationships that could be construed as a potential conflict of interest.

## Publisher's Note

All claims expressed in this article are solely those of the authors and do not necessarily represent those of their affiliated organizations, or those of the publisher, the editors and the reviewers. Any product that may be evaluated in this article, or claim that may be made by its manufacturer, is not guaranteed or endorsed by the publisher.

## References

[1] Serapioni M Ferreira S Lima TM. Voluntariado em Portugal: Contextos, Atores e Práticas. Fundação Eugénio de Almeida (FEA). (2013). p. 1–302.

[2] Abreu P. Volunteering in Portugal: Facts and Figures Report. Brussels: CEV. (2008)

[3] Almeida A Ferrão J Delicado A. Caracterização do Voluntariado Social em Portugal. Intervenção Social, (2002). p. 127–140.

[4] Jacinto LMJ. Evolução do voluntariado em portugal (2002-2020). Rev UI_IPSantarém - Unidade de Investigação do Instituto Politécnico fr Santarém. (2020) 8:157–68.

[5] Prisionais D-GdReS Justiça Md. Relatório de Atividades e Autoavaliações Atividades. (2019).

[6] PROACT. Estudo de Caracterização do Voluntariado em Portugal. Trabalho para o Conselho Nacional para a Promoção do Voluntariado; Unidade de Investigação e Apoio Técnico ao Desenvolvimento Local, À Valorização do Ambiente e à Luta contra a Exclusão Social. (2012). p. 59.

[7] Aproximar CdSS Straffälligenbetreuung VB. Prisons Managing Volunteers in Europe - an insight into prisons' perceptions, needs and current practices. (2020). p. 59.

[8] Uggen C Manza J Behrens A. ‘Less than the average citizen’: Stigma, role transition and the civic reintegration of convicted felons. In: After crime and punishment. (2013). p. 279–311.

[9] Chui WH Cheng KK. Effects of volunteering experiences and motivations on attitudes toward prisoners: evidence from Hong Kong. Asian J Criminol. (2012) 8:103–14. 10.1007/s11417-012-9148-9

[10] Chui WH Cheng KK. Self-perceived role and function of Christian prison chaplains and Buddhist volunteers in Hong Kong prisons. Int J Offender Ther Comp Criminol. (2013) 57:154–68. 10.1177/0306624X11432128 22186880

[11] Kerley KR Matthews TL Shoemaker J. A simple plan, a simple faith: chaplains and lay ministers in Mississippi prisons. Rev Relig Res. (2009) 51:87–103.

[12] Schuhmann C Kuis E Goossensen A. “Purely for You”: Inmates' Perceptions of Prison Visitation by Volunteers in the Netherlands. Int J Offender Ther Comp Criminol. (2018) 62:4545–64. 10.1177/0306624X18764523 29557241

[13] Kort-Butler LA Malone SE. Citizen volunteers in prison: bringing the outside in, taking the inside out. J Crime Justice. (2014) 38:508–21. 10.1080/0735648X.2014.969293

[14] Kjelsberg E Skoglund TH Rustad AB. Attitudes towards prisoners, as reported by prison inmates, prison employees and college students. BMC Public Health. (2007) 7:71. 10.1186/1471-2458-7-71 17480213 PMC1891097

[15] Brooker R Hu W Reath J Abbott P. Medical student experiences in prison health services and social cognitive career choice: a qualitative study. BMC Med Educ. (2018) 18:9. 10.1186/s12909-017-1109-7 29291725 PMC5748951

[16] Duncan HE Balbar S. Evaluation of a visitation program at a canadian penitentiary. Prison J. (2008) 88:300–27. 10.1177/0032885508319210

[17] Fernandes S. Manual de apoio na gestão de voluntariado. Porto: Federação Nacional de Associações Juvenis. (2016)

[18] Vicente P Gonçalves A Alves F Pinheiro H Barbedo M Branco R. Gestão do Voluntariado em Meio Prisional. (2009). p. 243.

[19] Lei 71/98 de 3 de Novembro. Diário da República. p. 5694–5696.

[20] Decreto-Lei 389/99 de 30 de Setembro. Diário da Assembleia da República. p. 6694–6698.

[21] Aproximar CdSS. How did volunteering shape prison life in 2020? 2020.

[22] Braun V Clarke V. Successful Qualitative Research: A Practical Guide for Beginners. London: Sage. (2013). p. 382.

[23] Tewksbury R Dabney D. Prison volunteers. J Offender Rehabil. (2004) 40:173–83. 10.1300/J076v40n01_09

[24] Clary EG Snyder M Ridge RD Copeland J Stukas AA Haugen J. Understanding and assessing the motivations of volunteers: a functional approach. J Personal Soc Psychol. (1998) 74:1516–30. 10.1037/0022-3514.74.6.1516 9654757

[25] Mears DP Cochran JC Siennick SE Bales WD. Prison visitation and recidivism. Just Quart. (2011) 29:888–918. 10.1080/07418825.2011.583932

[26] Cochran JC. The ties that bind or the ties that break: examining the relationship between visitation and prisoner misconduct. J Crim Justice. (2012) 40:433–40. 10.1016/j.jcrimjus.2012.06.001

