# Supplementary material for: The Experience of Volunteers in Prisons in Portugal: A Qualitative Study
Source: Front Psychiatry. 2022 Jan 4;12:778119. doi: 10.3389/fpsyt.2021.778119 (PMC8764396; doi:10.3389/fpsyt.2021.778119)
Supplement: Supplementary file 1 [file Data_Sheet_1.docx]

**Appendix 1 – Semi-structured interview guide** (adapted from Kort-Butler & Malone, 2014)

**Semi-structured interview guide**

***The Experience of Volunteers in Prisons in Portugal: A Qualitative Study***

This interview aims to study the experiences that volunteers have in the prison context during their volunteer work, as well as their perceptions of these experiences with inmates.

1. I would like to know your age and your profession.
2. How did you get involved in volunteering in prison?
3. What motivated you to volunteer in prison?
4. Describe some activities you have performed as part of volunteering.
5. How do you describe your interaction with inmates? Please don’t use names.
6. What do you consider most important in your relationship with inmates?
7. How do you describe your interaction with the professional staff?
8. In your opinion, what are the added value of volunteering in the prison context?
9. In your opinion, what are the challenges of volunteering in prisons?
10. What was your best experience as a volunteer? What was your worst experience?
11. Would you recommend that someone volunteer in prison? Why/Why not?
12. What recommendations would you like to make to improve volunteering in the prison context?
13. Would you like to add any more information?
