# Supplementary material for: The Experience of Volunteers in Prisons in Portugal: A Qualitative Study
Source: Front Psychiatry. 2022 Jan 4;12:778119. doi: 10.3389/fpsyt.2021.778119 (PMC8764396; doi:10.3389/fpsyt.2021.778119)
Supplement: Supplementary file 2 [file Data_Sheet_2.docx]

**Appendix 2 – Sociodemographic Questionnaire**

**Sociodemographic Questionnaire**

**Gender:** Female Male

**Age: ________**

**Marital status** (check with an X in the option that applies to you)**:**

Single Married Divorced Widower

**Educational qualifications** (check with an X in the option that applies to you)**:**

4^th^ year of schooling 9^th^ year of schooling High School

Bachelors’ degree Masters’ degree PhD degree

**Professional occupation: ________________________________________**

**Socioeconomic level** (check with an X in the option that applies to you)**:**

High Medium Low

**Volunteering time: _____________________**
