# Supplementary material for: The Experience of Volunteers in Prisons in Portugal: A Qualitative Study
Source: Front Psychiatry. 2022 Jan 4;12:778119. doi: 10.3389/fpsyt.2021.778119 (PMC8764396; doi:10.3389/fpsyt.2021.778119)
Supplement: Supplementary file 3 [file Data_Sheet_3.docx]

**Appendix 3 – Tables with themes and subthemes in Portuguese**

| Diferentes motivações para voluntariar | Interação dos voluntários com os reclusos | Interação dos voluntários com os profissionais da prisão | Voluntariado em contexto prisional causa impacto nos voluntários | Perceção dos voluntários sobre ajudar os reclusos | Maior apoio ao voluntariado em contexto prisional |
| --- | --- | --- | --- | --- | --- |
| Para ocupar o tempo  Fé religiosa  Necessidade de ajudar  Experiências anteriores de voluntariado  Recomendação de alguém  Oportunidade de voluntariar numa prisão surgiu | Interação positiva com os reclusos na prisão  Ganhar confiança com os reclusos  Melhorar a comunicação com os reclusos  Passar tempo fora da prisão durante as saídas de curta duração | Guardas prisionais inicialmente desconfiados dos voluntários  Voluntários vistos como obstáculos pelos guardas prisionais  Interação dos voluntários com os guardas prisionais melhorou com o tempo sendo vista como cordial  O ambiente prisional era difícil  Os técnicos gestores de voluntariado eram acessíveis com os voluntários | Mudou as perspetivas dos voluntários  Forçou os voluntários a gerir as suas expectativas  Relativização dos problemas dos voluntários | Aquisição de competências  Quebrar a rotina  Ponte entre os reclusos e as famílias  Vínculo social com o exterior | Proporcionar formação e acesso a apoio aos voluntários  Seleção cuidadosa das pessoas que voluntariam nas prisões  Melhorar as condições na prisão para a realização de atividades de voluntariado  Melhorar a relação entre as associações de voluntariado e os estabelecimentos prisionais  Melhorar a imagem da população reclusa na sociedade e promover a sua reintegração |

**Tabela 1|** Temas e subtemas.

**Tabela 2|** Citações sobre as diferentes motivações para voluntariar.

| Diferentes motivações para voluntariar | |
| --- | --- |
| **Para ocupar o tempo** | *"Reformei-me e tinha alguma disponibilidade. Como tinha tempo livre, acabei por ir a uma reunião inicial [...]" (Voluntário 39)*  "*[...] foi assim que comecei. Um pouco para ajudar, para ocupar o meu tempo em prol de algo maior*" *(Voluntária 10)* |
| **Fé religiosa** | *"O voluntariado em contexto prisional é uma consequência da minha fé católica" (Voluntário 16)*  *"[...]* *foi um pouco também por causa da minha religião porque eu tenho formação cristã*  *[...]" (Voluntária 10)* |
| **Necessidade de ajudar** | *"Sempre tive esta necessidade de querer ajudar outras pessoas" (Voluntária 37)*  *"Senti a necessidade de ter um compromisso complementar com a sociedade" (Voluntário 06)*  *"Pensar que poderia ajudar de alguma forma, isto é, que poderia dar um melhor contributo para dar às pessoas que estavam a passar por um momento de sofrimento" (Voluntária 02)* |
| **Experiências anteriores de voluntariado** | *"O voluntariado já [o] tinha começado mais cedo, mas noutro tipo de projetos" (Voluntária 15)*  *"O voluntariado sempre esteve comigo, e sempre voluntariei depois ao longo da minha vida" (Voluntária 31)*  *"O mundo das prisões sempre esteve presente na minha vida – a começar pelo meu pai*  *(foi médico na prisão durante muitos anos)*  *que contava histórias incríveis de casos de reclusos e depois o voluntariado que fiz quando tinha 18 anos, o que também me marcou muito." (Voluntária 01)* |
| **Recomendação de alguém** | *"É engraçado porque foi uma amiga que veio ter comigo e disse – olha, acho que tenho uma proposta de que* *vais gostar – [...] e como tinha um horário de trabalho flexível, decidi tentar." (Voluntária 17)*  *"Foi por sugestão de um amigo meu" (Voluntário 08)*  *"[...] Depois de me formar, fui trabalhar para o escritório de um advogado que era o líder de um grupo de visitadores* *no estabelecimento* *prisional*  *de Lisboa e ele convidou-me para participar naquele grupo."* *(Voluntário 29)* |
| **Oportunidade de voluntariar numa prisão surgiu** | *"Não tinha motivação [específica], era mais a de sair da minha zona de conforto" (Voluntária 09)*  *"Nunca me passou pela cabeça ir para o voluntariado prisional" (Voluntária 14)* |

**Tabela 3|** Citações das interações dos voluntários com os reclusos.

| Interação dos voluntários com os reclusos | |
| --- | --- |
| Interação positiva com os reclusos na prisão | *"A relação tem de ser baseada na verdade, na honestidade, sem paternalismo, sem estar de cima para baixo, o que me apercebi cedo e acho que sempre tentei ter isso, por isso acho que a relação foi sempre muito fácil, com igualdade de tratamento" (Voluntária 25)*  *"Uma atitude de honestidade, lealdade é necessária, não desmerecer a confiança que eles colocam em nós, nunca de forma alguma." (Voluntário 33)* |
| Ganhar confiança com os reclusos | *“[…] um recluso que disse que nós fomos muito importantes porque indo lá todas as semanas nós mostramos que tínhamos confiança nele e ele disse que os presos não tinham confiança em ninguém, nem neles nem em ninguém […]” (Voluntária 07)*  *“As conversas que tenho com eles, muitas vezes eu conto-lhes os meus defeitos, isso dá-lhes essa confiança de que eles não são anormais mas sim pessoas que foram mais além do que era suposto ir, mas pronto a partir de agora, é pegar nisso e transformar e depois eles começam a ter esta conversa.” (Voluntário 33)* |
| Melhorar a comunicação com os reclusos | *“Se há questões, quer dizer aquilo é sempre explosivo porque estamos sempre a encontrar pessoas com características muito diferentes e que são obrigadas a conviver naqueles espaços e naquelas condições, portanto eu acho que a calma é uma coisa que não habita dentro destes espaços, mas nós pelos menos quando lá estamos, tentamos que os pensamentos voem para outros lados […]” (Voluntária 31)*  *“A relação que se estabelece é portanto uma relação de conhecimento, é uma pessoa que nos é apresentada e a quem nos apresentamos e, a partir daí começamos uma conversa que é marcada pelo espaço da visita solidária é um espaço de liberdade por essencial […]” (Voluntário 22)* |
| Passar tempo fora da prisão durante as saídas de curta duração | *"Eu faço as saídas precárias. [...] Basicamente, somos responsáveis por aqueles que têm o direito de fazer estes passeios precários, apanhamo-los e saímos uma tarde com eles, almoçamos e ficamos até meio da tarde com eles. [...] Temos uma grande ligação, conhecemo-los há alguns anos." (Voluntário 04)* |

**Tabela 4|** Citações das interações dos voluntários com os profissionais da prisão.

| Interações dos voluntários com os profissionais da prisão | |
| --- | --- |
| Guardas prisionais inicialmente desconfiados dos voluntários | *"O início foi um pouco perturbado porque eram pessoas muito desconfiadas, frias e um pouco rígidas" (Voluntária 38)*  *"No início eram muito desconfiados [dos voluntários]" (Voluntário 39)* |
| Voluntários vistos como obstáculos pelos guardas prisionais | *"[...] mas na maioria deles, vou ser honesto consigo, o que nos passa é que eles não nos veem como uma mais-valia, é quase mais um obstáculo." (Voluntária 10)*  *"[...] ter uma relação com eles que ajuda a desfazer esta ideia do corpo estranho, mas alguns têm dificuldade em empatia, alguns são mais fáceis" (Voluntário 03)* |
| Interação dos voluntários com os guardas prisionais melhorou com o tempo sendo vista como cordial | *"Houve uma evolução muito interessante. Até numa primeira fase, nós víamos os guardas quase como um obstáculo de acesso aos reclusos e eles também nos viam com algum desdém, com alguma reserva. Depois, percebemos que quando nós vamos visitar todos os que estão na prisão, nós vamos visitar os guardas, os auxiliares com quem nos cruzamos, e todos eles. […] Portanto, nós vamos visitar o meio prisional, vamos visitar os reclusos, vamos visitar os guardas que os protegem e toda a gente que está lá incluída, e isso modificou completamente a relação. Com o tempo, foi-se modificando [a relação com os guardas prisionais].” (Voluntário 06)*  *“A minha relação com os guardas é de mútuo respeito.” (Voluntária 34)*  *“A nossa relação tenta ser o mais cordial e correta possível, tentamos ser próximos deles” (Voluntário 03)* |
| O ambiente prisional era difícil | *“A que mais me impressionou foi […] uma cadeia [especial] de alta segurança, em que os presos estão fechados 23h, não se ouve uma mosca, é uma coisa horrível. Eu fazia entrevistas com os presos para os convencer a fazerem trabalhos para a exposição, só 1 é que acedeu e aí também tive uma reunião com ele sozinha, mas aí foi um bocado complicado porque meteram-me numa sala com ele e depois aquilo só se pode sair quando se aciona um botão. Portanto, aquilo foi um bocado tenso.” (Voluntária 11)*  *“Eu estava com eles [reclusos] numa sala onde caía humidade, […] escorria água pelas paredes, portanto este não é um ambiente aprazível, digamos […]. Eu acho que devia de haver salas para estar com as pessoas, eu próprio não tirava o sobretudo lá dentro. Era um gelo completo.” (Voluntário 19)*  *“[…] a vida dentro do estabelecimento prisional é horrível. É horrível, olhe os corredores da cadeia […], são imensos, larguíssimos, altos e nos dias de inverno o nevoeiro que está cá fora, está lá dentro; a humidade que está cá fora, está lá dentro […]” (Voluntário 03)* |
| Os técnicos gestores de voluntariado eram acessíveis com os voluntários | *"Com as técnicas, com uma ou outra, a amizade até foi criada, mas eu sempre na linha de cá. Foi criada uma relação de amizade" (Voluntária 35)*  *"Também tenho uma relação positiva com os técnicos." (Voluntária 37)*  *"Com os educadores técnicos, diretor adjunto e diretor num clima de confiança e cordialidade" (Voluntário 33)* |

**Tabela 5|** Citações do impacto que o voluntariado em contexto prisional causa nos voluntários.

| Voluntariado em contexto prisional causa impacto nos voluntários | |
| --- | --- |
| Mudou as perspetivas dos voluntários | *"Percebo que a minha realidade não é a única e é sempre conhecida tanto como qualquer experiência fora do seu contexto. É por isso que acho que é isso, dá maior abertura social, estou mais consciente das realidades que existem e das situações de injustiça que também existem" (Voluntária 25)*  *"Cada contacto com uma realidade diferente da nossa ajuda-nos a criar a possibilidade de empatia e, não sei, abre um pouco do mundo e das nossas cabeças para compreender outras realidades" (Voluntária 25)* |
| Forçou os voluntários a gerir as suas expectativas | *"Aquela expectativa de se for possível colaborar para que uma pessoa se reintegre na sociedade, estamos sempre com essa expectativa, embora não seja isso que esperamos" (Voluntariado 08)*  *"[…] ir e não esperar por nada, ir e unicamente para estar com eles e mais nada. [...] Não estar à espera de nada deles, mas dar-lhes uma manhã diferente" (Voluntária 07)* |
| Relativização dos problemas dos voluntários | *"Relativizamos muito mais certas coisas que nos acontecem na vida" (Voluntária 26)*  *"Colocamos as coisas na prioridade certa. [...] Damos mais valor ao que temos e ao que normalmente damos por garantido" (Voluntária 15)* |

**Tabela 6|** Citações da perceção dos voluntários sobre ajudar os reclusos.

| Perceção dos voluntários sobre ajudar os reclusos | |
| --- | --- |
| Aquisição de competências | *"Aliviar a tensão, estar ocupado. Alguns aprendem profissões e como ser útil à sociedade através dos contactos dos workshops que vendem o que estão a produzir." (Voluntária 34)*  *"As ferramentas ajudam a estabelecer o diálogo, a partilhar ideias, e até a se conhecerem melhor" (Voluntário 28)*  *"Tentámos levar algumas atividades variadas, desde textos a algo mais prático para eles também, para que eles participassem [...]" (Voluntária 24)*  *"Sempre preparámos um tema, um texto, uma dinâmica para os envolver e ajudá-los a partilhar, mas a conversa individual também é muito importante." (Voluntária 23)* |
| Quebrar a rotina | *"Ajuda passar o tempo e é construtivo, são coisas construtivas. Habituam-se a estar num grupo, a ter horários, a ter disciplina. As rotinas do dia-a-dia e as semanas de alguma forma, os nossos projetos estavam lá a quebrar algumas rotinas" (Voluntária 17)*  *"É importante contribuir para fazer um pouco da diferença nos dias deles" (Voluntária 37)* |
| Ponte entre os reclusos e as famílias | *“Muitas vezes acabamos por fazer o contacto com as famílias e levar, ou ajudar, familiares a visitar […] e isso acontece, às vezes nós patrocinamos a vinda de uma família […] da Guarda ou de outro ponto do país, para que possam vir visitar a reclusa que está em Tires” (Voluntária 17)*  *“Nunca tive medo porque não tenho motivos, […] há uma conversa de continuidade, geralmente recolhemos números de telefone para telefonar às famílias.” (Voluntária 14)*  *“Fazemos um bocadinho esta ponte entre a reclusa que está lá dentro e a família que está cá fora e isso também é muito gratificante e é uma coisa que não nos custa nada. Tudo aquilo que nós podermos fazer e que seja o básico e inofensivo, nós tentamos ajudar sempre com o conhecimento da cadeia” (Voluntária 01)* |
| Vínculo social com o exterior | *"Somos alguém que vem de fora e traz algo novo. [...] É* *importante que tenham alguém* *com quem conversar, alguém fora do sistema" (Voluntária*  *02)*  *"Somos um pouco a janela que se abre para eles, a janela que vem de fora e trazemos um pouco de encorajamento, de esperança, de confiança" (Voluntária 01)*  *"[...] a fim de lhes dar algum convívio, alguma coexistência com o mundo exterior que eles não tinham, nem a família os visitou" (Voluntário*  *08)* |

**Tabela 7|** Citações sobre maior apoio ao voluntariado em contexto prisional.

| Maior apoio ao voluntariado em contexto prisional | |
| --- | --- |
| Proporcionar formação e acesso ao apoio aos voluntários | *"As formações acho que são muito importantes, que é dar-nos a força para ir, acreditar, sentirmo-nos renovados na ajuda" (Voluntária 14)*  *"A melhor forma de melhorar a atividade de voluntariado é manter a formação crítica e contínua [...]" (Voluntário 06)*  *"Foi mais o apoio que nos dão do estabelecimento. Acho que deles não temos tanto apoio como devíamos ter" (Voluntária 10)* |
| Seleção cuidadosa das pessoas que voluntariam nas prisões | *"Eu recomendo o voluntariado em contexto prisional apenas para pessoas que têm um conjunto de características muito específicas. [...] É preciso ser um indivíduo persistente, motivado, com uma capacidade extraordinária de ouvir." (Voluntário 22)*  *"O voluntário tem que ter certas características muito fortes para enfrentar tal*  *desafio. Acima de tudo, saber ouvir, não fazer julgamentos, [...] dar opinião quando necessário, manter o sigilo absoluto,* *não entrar em campos jurídicos, parecem coisas muito simples, mas não o são para muitas pessoas" (Voluntária 35)*  *"Tens de ter um perfil, sabes?" Tendemos a aceitar pessoas já com alguma motoridade, que não se deixem envolver emocionalmente com os reclusos, que são cúmplices, que são*  *fiéis,*  *não estamos propriamente a fazer um trabalho que qualquer um possa fazer.* *" (Voluntário 06)* |
| Melhorar as condições na prisão para a realização de atividades de voluntariado | *"[...] em termos de instalações para o desempenho das atividades, deve, por conseguinte, existir um esforço institucional da Direção-Geral para criar, dentro das possibilidades físicas, condições para que este voluntariado possa ser feito de forma mais profícua.* *O voluntariado [...] é uma realidade externa,*  *tem de se adaptar e as adaptações e os ajustes têm de ser feitos, e há coisas que às vezes beneficiariam se pudessem ser feitas no seu próprio espaço e com condições adequadas para que haja uma diferenciação física, que seja um espaço de liberdade dentro de um espaço de*  *reclusão.* *" (Voluntário 22)* |
| Melhorar a relação entre as associações de voluntariado e os estabelecimentos prisionais | *"Estou convencido de que a relação com entidades do sistema prisional é importante no voluntariado em contexto prisional. [...] ser capaz de quebrar esta barreira, no sentido de criar um bom ambiente entre entidades prisionais e trabalho voluntário, que eu acho que era algo a ser feito. Esta*  *relação com a estrutura prisional é importante*- *seria esse o conselho que daria - apostar na relação com a estrutura* *prisional." (Voluntário 08)*  *"Acho que o voluntariado deve acabar com as "capelinhas", não deve haver "capelinhas", sim, tenho a minha organização e vocês têm a vossa. Este nível de ajuda mútua entre associações, na forma como eu vejo, não existe. Se houvesse uma união dos voluntários [...] talvez pudéssemos mudar certas* *regras para que pessoas mais dignas, mais humanas, viessem a público." (Voluntário 33)*  *"Uma maior flexibilidade em termos de acreditação, o processo de admissão de voluntários e colaboradores assistentes espirituais que não são realmente visitantes voluntários é muito moroso e isso é por vezes desencorajador.* *" (Voluntário 22)* |
| Melhorar a imagem da população reclusa na sociedade e promover a sua reintegração | *"Gostaria muito que o sistema prisional olhasse para o voluntariado como um veículo de reintegração. [...] gostava que o voluntariado, no geral, fosse visto como mais um braço para ajudar estas pessoas com a sua reintegração e às vezes nem sequer é reintegração, é integrarem-se pela primeira vez na vida" (Voluntária 31)*  *"Voluntariado estendido para pós-prisão" (Voluntária 02)*  *"Atendendo à realidade prisional, é muito importante que isso aconteça e que haja interação entre a sociedade civil [não reclusa] e a sociedade reclusa porque é realmente uma parte da população que está totalmente isolada e não se tem [contacto], pelo menos nunca tive contacto com ela, é uma realidade completamente desconhecida do normal, por isso é inevitável que o estigma dure para sempre e que uma pessoa saia e não tenha oportunidades." (Voluntária 25)* |
